# Ecology and evolution of antimicrobial resistance in bacterial communities

**DOI:** 10.1038/s41396-020-00832-7

**Published:** 2020-11-20

**Authors:** Michael J. Bottery, Jonathan W. Pitchford, Ville-Petri Friman

**Affiliations:** 1 Department of Biology, University of York, Wentworth Way, York, YO10 5DD UK; 2 Department of Mathematics, University of York, Heslington, York, YO10 5DD UK

**Keywords:** Microbial ecology, Antibiotics, Bacterial evolution

## Abstract

Accumulating evidence suggests that the response of bacteria to antibiotics is significantly affected by the presence of other interacting microbes. These interactions are not typically accounted for when determining pathogen sensitivity to antibiotics. In this perspective, we argue that resistance and evolutionary responses to antibiotic treatments should not be considered only a trait of an individual bacteria species but also an emergent property of the microbial community in which pathogens are embedded. We outline how interspecies interactions can affect the responses of individual species and communities to antibiotic treatment, and how these responses could affect the strength of selection, potentially changing the trajectory of resistance evolution. Finally, we identify key areas of future research which will allow for a more complete understanding of antibiotic resistance in bacterial communities. We emphasise that acknowledging the ecological context, i.e. the interactions that occur between pathogens and within communities, could help the development of more efficient and effective antibiotic treatments.

## Introduction

The global use and misuse of antibiotics has led to the evolution and spread of bacterial resistance to all routinely used antibiotics. In order to effectively tackle the spread of antimicrobial resistance (AMR) through antimicrobial stewardship or development of novel antimicrobial compounds, understanding how bacteria adapt and evolve to survive antibiotic treatments is crucial. Here we argue that to fully understand the evolution of AMR we must study the emergence of resistance within the context of microbial communities. While the composition, structure and interactions within microbial populations are known to affect the horizontal gene transfer (HGT) of antibiotic resistance [1–3], it is becoming evident that intra- and interspecies interactions also influence how species respond and evolve under antibiotic exposure within microbial communities. As a result, clinical susceptibility to antibiotics might not translate well to the successful treatment of polymicrobial infections where pathogens are typically embedded in complex multispecies microbial communities. Interspecies interactions are not typically accounted for when assessing antibiotic sensitivity and resistance evolution in the context of antibiotic treatments. In this perspective, we focus on the role of interspecies bacterial interactions on the selection of pre-existing resistance, and on how interactions within bacterial communities could change the evolutionary trajectory of de novo resistance, i.e., the evolutionary dynamics and outcomes of antibiotic exposure, particularly within a clinical context.

The ability of a bacterial strain to survive antibiotic exposure is commonly assessed in monoculture measurements of growth, through broth microdilution or agar dilution assays, to quantify a minimum inhibitory concentration (MIC) [4]. These in vitro methods test the susceptibility of individual bacterial strains determining MIC breakpoints and providing informed choices of antimicrobial interventions. However, our understanding and interpretation of MIC measurements has improved in recent years. Higher initial population densities can result in lower susceptibility to some antibiotics, a phenomenon called the inoculum effect [5, 6]. In addition, biofilm growth can dramatically increase the effective MIC of a population and alter the rates of resistance evolution when compared to planktonic cells [7, 8]. Finally, it is increasingly evident that sub-MIC antibiotic concentrations can positively select for resistance mutations, increase HGT of antimicrobial resistance genes (ARGs), and elevate mutation rates; all of which will increase the likelihood of resistance evolution [9–12]. As such the minimal selective concentration (MSC) and predicted no effect concentration have become important metrics for understanding and controlling the evolution and spread of resistance [13].

The effect of ecological context, i.e., the type and dynamics of microbial interactions that occur between pathogens and the surrounding community, is often overlooked when considering antibiotic efficacy and the evolution of resistance. We currently predominantly consider antibiotic sensitivity as an independent trait of an individual strain. However, it is clear that inter- and intra-species interactions within both clinical and environmental communities, as well as the physical properties of the associated microbial community, can alter the physiological state and antibiotic susceptibility of bacteria [14]. Interspecies interactions are especially common in polymicrobial infections where multiple species contribute to disease [15]. These interactions not only affect the severity of infections but could also alter the exposure and physiological responses to antibiotics. We thus argue that bacterial ability to withstand antibiotic treatments, and the consequent evolution of resistance to those treatments, should be viewed as an emergent property of a microbial community rather than simply a trait of a particular species.

To discuss the effects of antimicrobial exposure on microbial communities we must first distinguish the different ways in which bacteria survive in the presence of antibiotics. Resistance is often defined as the inheritable ability of a cell to grow at high concentrations of an antibiotic, irrespective of the duration of exposure [16]. Its mechanisms are many and varied [17]. Resistance can be intrinsic, whereby the physiological properties of all members of a species allow it to resist the action of certain antibiotics; or acquired, either through de novo mutation or via the acquisition of ARGs via HGT. However, bacterial populations can also survive antibiotic treatment without encoding specific resistance mechanisms. Tolerance describes the ability of a bacterial population to survive transient exposure to a high concentration of an antibiotic without a change in their MIC [16]. This is often mediated by a change in the physiological state of the cells within a population due to their environmental context, such as slowing down essential cellular processes following a lack of resources [18], interactions with a host’s immune system [19] or interactions with other bacterial species [20], resulting in a reduced death rate in the presence of antibiotic. Alternatively, tolerance can be a heritable and evolvable trait, as has been demonstrated in the evolution of tolerance through the optimisation of lag-times to match the intervals of antibiotic exposure [21]. Whereas resistance and tolerance are considered properties of a population, persistence describes the ability of a small sub-population of a clonal bacterial population to enter a state of dormancy, allowing it to survive high concentrations of antibiotic while the rest of the population is rapidly killed [22, 23]. Both tolerance and persistence allows surviving bacteria to resume normal growth after the depletion of the antibiotic.

## Interspecies interactions can alter responses to antibiotics

Interspecies interactions can have a profound effect upon the outcome of antibiotic treatment. We define three main ways in which bacterial communities, or subpopulations of communities, can survive exposure to antimicrobials due to interspecies interactions: (1) *Collective resistance*, interactions within a community that elevate the ability of its members to resist the action of an antibiotic and continue to grow in the presence of antibiotics thus increasing the MIC of the community. (2) *Collective tolerance*, interactions within a community that alter cell state, such as slowing down metabolism, and thus slow down the rate of cell death during transient exposure to antibiotics without an increase in MIC. (3) *Exposure protection*, interactions within a community that protect its sensitive members during antibiotic treatment by reducing the effective concentration of antibiotic [24, 25]. These definitions are not mutually exclusive; interactions could have multiple different effects, and multiple different mechanisms may act simultaneously within a community.

Many of the known mechanisms that provide collective resistance, tolerance or exposure protection are density-dependent, providing protection to large, dense populations [25] through the inactivation of antibiotics, biofilm formation and quorum sensing-activated responses [26–28]. While such interactions are often considered to take place among cells of the same (or closely related) genotypes, the same mechanisms can also occur between different genotypes or species in diverse multi-species communities. For example, an analysis of the multi-species networks of urinary tract infections showed that clinical isolates from the same patient often protect each other from antibiotics, with the protective effects correlating with interspecies interactions that foster growth benefits [29]. However, the majority of interactions within multispecies communities are argued to be competitive, with most pairwise interactions resulting in a net reduction in community productivity [29, 30]. Disruption of the complex network of interactions within communities, whether cooperative or competitive, through exposure to antimicrobials will thereby alter community structure and change the growth and survival of its constituent members.

**Fig. 1:**
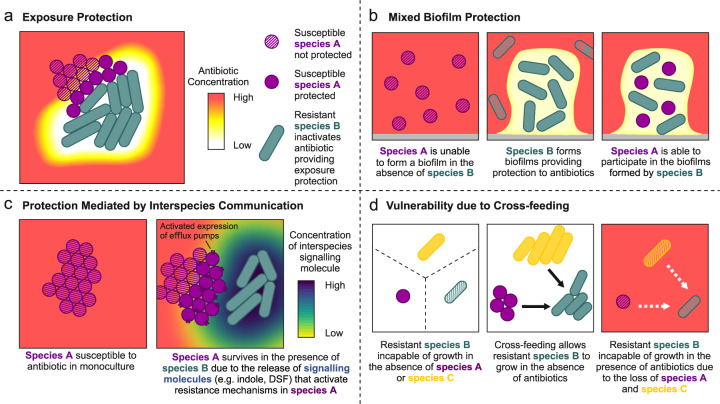
Community interactions, as well as resistance genotype, affect the response to antibiotic exposure. In all panels, cell growth state is represented by either hatched (unable to grow) or solid fill (able to grow). **a** Resistant bacteria that inactivate antibiotics reduce the local antibiotic concentration, providing exposure protection to surrounding sensitive species. This benefit is dependent upon the density and spatial structure of the population, as well as the diffusion rate and rate of inactivation of the antibiotic. **b** Some species are unable to form biofilms in isolation but are able to gain improved antibiotic tolerance by participating in established biofilms of other species. **c** The receipt of secreted signalling molecules, such as indole and DSF, can trigger antibiotic resistance states in otherwise susceptible community members through increasing the expression of resistance genes. **d** Reliance upon cross-feeding networks can be detrimental for a resistant species if its growth depends on cross-feeding on secretions of susceptible community members. In this scenario, tolerance to antibiotics is lowered to the level of the most susceptible community member as cross-feeding interactions are lost due to antibiotic killing (dashed white arrows) thus the resistant species is unable to grow due to the loss of essential recourses.

Resistance mechanisms that inactivate antibiotics are widespread, providing both acquired and intrinsic resistance to some of the most widely used antibiotics including aminoglycosides, chloramphenicol and β-lactams. Such resistance mechanisms can be considered as cooperative traits; the benefit of antibiotic inactiaving enzyme production is not confined to the producer, rather it is shared across the community [31, 32]. Cooperative enzymatic inactivation provides exposure protection through reducing the environmental concentration of an antibiotic (Fig. 1a), which can be exploited by sensitive members of the same, and different, species within a community [26, 33–35]. For example, the enzymatic inactivation of β-lactam antibiotics via β-lactamase producing *Escherichia coli* can protect *Salmonella enterica* serovar Typhimurium from concentrations of antibiotic that would usually kill them [33]. The presence of β-lactamases has been reported in a wide range of clinical samples taken from polymicrobial infections (reviewed in [36]) suggesting that they likely contribute to the resilience of infections to β-lactam antibiotics. The benefit of antibiotic inactivation is not limited to periplasmic or secreted enzymes; the intracellular inactivation of chloramphenicol by *Staphylococcus aureus* or *Streptococcus pneumoniae*, via a plasmid borne acetyltransferase, allows the survival of sensitive *S. pneumoniae* through the detoxification of the local environment in liquid culture, on semi-solid surfaces and in in vivo mouse models [26]. Computational modelling suggests that the benefits of such cooperative antibiotic inactivation is greatest when community interactions are mutualistic, as mutualism drives the spatial mixing of the communities and hence the sharing of the benefit of antibiotic inactivation [37]. As a result, antibiotic inactivation could provide protection to a microbial community allowing sensitive members of the community to survive otherwise lethal antibiotic treatments.

Collective resistance, collective tolerance and exposure protection to antibiotics can also be provided through biofilm formation (Fig. 1b). Bacterial biofilms comprise of cells growing as aggregates surrounded by a self-secreted polymer matrix of exopolysaccharides, DNA and proteins. Biofilms provide exposure protection to their members through limiting the diffusion of antibiotics into the population [38] and increasing the protection provided by antibiotic inactivation [34]. In addition, biofilms can induce tolerant cell states due to nutrient and oxygen gradients that lead to a reduction in metabolic activity in the centre of the biofilm [27], increasing the proportion of persister cells within the population [23]. Biofilms can also increase levels of resistance by altering the expression of pre-existing ARGs [39]. Compared to single-species biofilms, interspecies interactions within multi-species biofilms can lead to further increases in survival during antibiotic treatment through altering spatial structure of the biofilm, increasing the expression of resistance mechanisms, and allowing for individually expressed antimicrobial defences to protect the whole community [40–42]. However, competition between genotypes via the secretion of toxins [43–45] and the seizing of space via the use of adhesins [46, 47] and matrix production [48] may limit social mixing of different genotypes within a biofilm and eradicate competing species [49]. Thus, competition may help to privatise public goods within multi-species biofilms, limit between-genotype cooperation and break down interactions that increase the ability of the community to survive antibiotic treatment.

Much work on biofilms has focused on the interspecies interactions within the lungs of Cystic Fibrosis (CF) patients, where multi-species biofilm formation is common. Within mixed biofilms *Pseudomonas aeruginosa* can cause a metabolic shift in *S. aureus*, reducing its growth and providing *S. aureus* with protection to vancomycin [50]. Reciprocally, *S. aureus* can enhance tobramycin tolerance of *P. aeruginosa* by increasing aggregation and altering biofilm architecture in CF model systems [51]. However, the activity of antibiotics against multi-species biofilms appears to depend on both community composition and the antibiotic treatment, with some combinations of bacterial species decreasing rates of killing by antibiotics, while others had no effect [52]. Whether interactions within biofilms facilitate or limit antibiotic resistance or tolerance [53] might depend on the mode of the interaction*,* i.e., direct cell contact versus diffusible molecules, as the physical properties of the community alter the probability of cell-cell contact and diffusion rates.

Bacteria secrete a vast variety of compounds ranging from signalling molecules involved in quorum sensing to exotoxins involved in virulence and competition. Secreted products within communities have been shown to directly alter gene expression, metabolic processes and the growth dynamics of co-residing bacterial species, altering their ability to survive antibiotic exposure (Fig. 1c). For example, interspecies signalling via the secretion of indole by *E. coli* activates the expression of an indole-dependent multi-drug efflux pump in *Pseudomonas putida*, which cannot produce the indole itself, leading to elevated levels of resistance in *P. putida* [54]. Likewise, the secretion of diffusible signal factor by *Stenotrophomonas maltophilia*, a Gram-negative bacterium that often co-occurs with *P. aeruginosa* during polymicrobial pulmonary infection, alters *P. aeruginosa* biofilm structure and stimulates the synthesis of proteins that provide resistance to cationic antimicrobial peptides such as polymyxin [55]. In addition to cellular cross talk, exotoxins — presumably produced to inhibit the growth of competitors — can promote the transition of competing bacterial species into an antibiotic tolerant physiological state. For example, *P. aeruginosa* induces highly tolerant small colony variants of *S. aureus* through the secretion of 4-hydroxy-2-heptylquinoline-*N*-oxide, which inhibits the electron transport chain, dramatically slowing down the growth rate of *S. aureus* [20, 56]. Given the vast diversity of bacterial secreted products, and the complexity of multi-species communities, it is likely that we have barely scratched the surface of understanding how these secretions alter antibiotic resistance and tolerance.

Competitive interactions, via interference or exploitation, are prevalent within mixed bacterial communities due to overlaps in metabolic requirements and limitation of space [29, 30, 57]. Disruption or alteration in the intensity of interspecies competition, or cooperation, through exposure to antibiotics can alter community structure by making otherwise inaccessible resources available for exploitation by other members of the community. These changes in community structure may alter the ability of component members to survive antibiotic exposure. Antibiotic treatment of brewery multi-species biofilms, which were dominated by competitive interactions, resulted in reduced levels of competition due to the inhibition of highly competitive species within the population. This led to increased growth of species that were otherwise suppressed within the population [58] and in turn elevated the antibiotic tolerance of these species, likely due to the density-dependent nature of collective tolerance mechanisms. Similar results were observed within experimental bacterial communities [59], where antibiotic treatment benefited members of the community that had marginally elevated levels of tolerance. In contrast, the mutualistic interaction of cross-feeding can lead to inter-dependence between multiple species within a community; thus lowering the antibiotic sensitivity of all members of the cross-feeding network to that of the weakest member of the consortium (Fig. 1d) [37, 60]. Taken together, these findings suggest that how individuals and communities respond to antibiotic exposure depends on a complex network of interactions including social exploitation, cooperation, competition and communication.

## Ecological context can influence the selection for antimicrobial resistance

How does a multi-species community alter the selection for AMR within its members? The answer to this question depends upon the members of the community in question and the specific interactions that occur between them. Multiple interactions — including competition, exploitation, commensalism and mutualism — take place in communities and each of them has the potential to alter the strength of selection acting upon resistance mechanisms of its members. Consequently, AMR evolution within communities may occur at different rates, result in different magnitudes of resistance, or be associated with different levels of cost compared to single species populations. The presence of interacting species could further affect the evolutionary trajectory of de novo resistance by changing which mutations and molecular mechanisms are selected for.

**Fig. 2:**
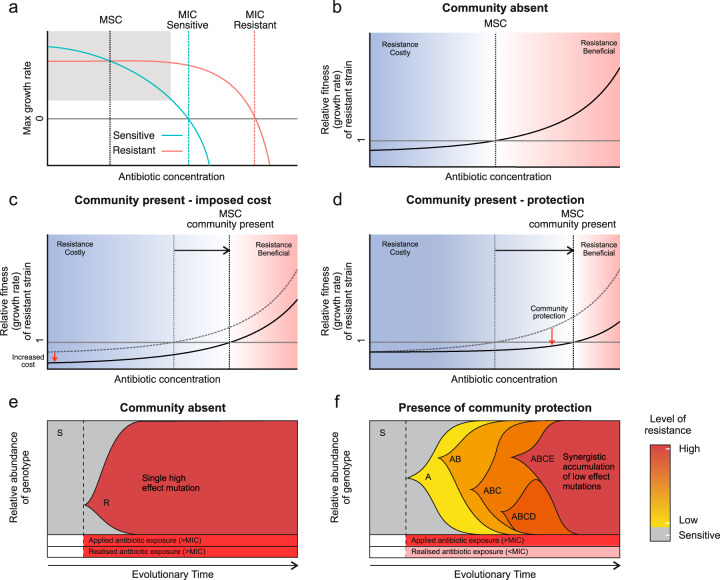
Fitness and selection consequences of differential effects of antibiotic concentration on growth rate. **a** Theoretical max growth rates in pure culture of ‘isogenic’ strains differing only in resistance or sensitivity to antibiotic. Shaded area represents the range of antibiotic concentrations in subsequent panels (**b**–**d**) exploring competition outcomes and minimal selective concentration (MSC) based on these relative growth rates. **b** The MSC is defined as the antibiotic concentration at which growth rate of the resistant strain exceeds that of the sensitive strain (relative fitness >1). **c, d** In a community, the MSC can be increased by two basic mechanisms. One is increased costs of resistance, which may arise by increased competition for nutrients (**c**) and the other is reduced antibiotic effect upon sensitive strains, which may arise by community protection (**d**). **e**, **f** The emergence of de novo resistance mutations can be altered by community protection. **e** In the absence of a protective community antibiotic exposure above the MIC acts upon standing genetic variation, often selecting for a single high resistance, high cost mutation. **e** Communiti**e**s that provide exposure protection may reduce the realised antibiotic exposure to sub-MIC levels, allowing for the sequential accumulation of low cost, low resistance mutations that together provide high levels of resistance for example via epistasis. Letters inside panels (S, R, A, AB, etc.) represent the accumulation of different mutations during selective sweeps.

Previous studies of the evolutionary dynamics of ARGs have mostly focused on resistant and sensitive strains within single-species populations [9, 32, 61]. These studies indicate that the concentration of antibiotic required to positively select for resistant bacteria, MSC, is far below the MIC of their sensitive counterparts [9, 61]. Low antibiotic concentrations at the MSC may be non-lethal to the sensitive strains, but they reduce the growth rate of sensitive cells sufficiently to outweigh the inherent costs associated with harbouring resistance [62–64]. Low levels of antibiotics could thus already select for an increase in the abundance of antibiotic resistant cells within a population (Fig. 2a/b). There are few experimental studies comparing how antibiotic resistant and sensitive strains respond to antibiotic selection in the absence and presence of other competing bacteria. Klümper et al. [14] recently demonstrated that complex multi-species anaerobic communities derived from pig faeces can increase the concentration of antibiotics required to select for resistant *E. coli* strains embedded within the community. Mechanistically, it was shown that this increase in MSC was caused either by protection of the sensitive *E. coli* strain by the community (Fig. 2d) (as previously shown with cooperative antibiotic inactivation in single species populations [31, 32]) or through competition elevating the magnitude of costs associated with the resistance (Fig. 2c) with no resulting change in the MIC of the sensitive strain [14]. More generally, these results are in line with previous studies showing that competition can constrain bacterial adaptation [65] supporting the idea that antagonistic bacteria-bacteria interactions are likely to limit the evolution of antibiotic resistance in communities.

Interactions within a community that increase the probability of the survival of its sensitive members during antibiotic exposure will also alter the selection dynamics of ARGs within the community [31, 32]. When a resistant strain provides protection to an otherwise sensitive strain within a community, the MSC of resistance would be expected to increase (Fig. 2d). This is because sensitive strains would be able to outcompete resistant strains at relatively higher concentrations of antibiotic [66]. However, the selection dynamics of resistance would typically depend upon the nature of protection provided by the community. When protection is provided by a single resistant species, for example through exposure protection via antibiotic inactivation, selection for this resistant genotype would be expected to follow negative frequency dependence. When the resistant strain is rare exposure protection will be preferentially directed towards the resistant genotype. As the resistant strain becomes more common, exposure protection will be shared with a larger proportion of sensitive members of the community and thus the relative fitness of the resistant genotype will decrease. In addition, if protection is provided by a single keystone species, protection might be more vulnerable to stochastic events and density fluctuations of the protective species. In contrast, if protection is provided by multiple species present in the community, or by the physical properties of the community (e.g., biofilm matrix), collective resistance or tolerance would likely be more stable due to relatively higher functional redundancy of “protection” within the community. Ecological stability of collective resistance and collective tolerance could thus alter the length of antibiotic exposure, and therefore alter the timescales in which selection for resistance can act [67, 68]. Inactivation of antibiotics may result in transient and short-lived antibiotic exposure leading to changes at ecological rather than evolutionary timescales, that is, changes in the composition and frequencies of different species within the community similar to the effect of ecological disturbances. In contrast, interspecies interactions that increase antibiotic tolerance may prolong antibiotic exposure at sub-inhibitory concentrations, allowing the accumulation of de novo mutations upon which selection can act [69] (in contrast to >MIC selection which typically acts upon the standing genetic variation within the population [Fig. 2e] [11]). Thus, communities could alter the trajectory of resistance evolution, allowing the stepwise generation of low effect, low cost resistance mutations that together provide high levels of resistance (Fig. 2f). Similar results have been observed during sub-MIC selection for resistance in *S. enterica*, which resulted in changes in multiple different resistance mechanisms at the molecular level, including alteration of the ribosomal RNA target, reduction in antibiotic uptake and induction of antibiotic-modifying enzymes [11].

Interspecies competition inherent to complex microbial communities can also elevate the magnitude of cost of resistance [14, 70], which may favour the selection for low cost antibiotic resistance mutations, or alternatively, increase the likelihood of compensatory mutations that overcome the associated costs of resistance. Similar trade-offs between cost and resistance have been observed when comparing the evolution of resistance between planktonic and biofilm lifestyles [71], with members of biofilms acquiring low cost mutations that provided overall lower levels of resistance compared to resistance mutations acquired during planktonic growth. Moreover, it has recently been shown that bacterial resistance evolution to phage can be changed in the presence of competitors from more costly surface receptor-mediated resistance to less costly CRISPR-based resistance [70]. While antibiotic and phage resistances are not directly comparable, both are mediated by multiple different mechanisms with varying magnitudes of cost, and their evolutionary trajectories could thus be similarly shaped by the presence of competitors. More experimental studies are needed to better understand the multiple ways interacting species might affect antibiotic resistance evolution in microbial communities.

## Significance of interspecies interactions for the success of antibiotic treatments

Even though interspecies interactions during polymicrobial infections — as well as between focal pathogens and resident microbiota of the body, such as those found in the oral cavity or gastrointestinal tract [72, 73] — are known to alter the outcome of antibiotic treatments [74, 75], these interactions are often overlooked. For example, relying on testing the antibiotic susceptibility of pathogens in monocultures could confound our ability to select the most appropriate antibiotics for clinical interventions because these tests might not reliably reflect community-scale responses. Indeed, in vitro antibiotic susceptibility does not always translate to successful treatment of polymicrobial infections, where disease is caused by multiple interacting pathogens [76]. In fact, the response of individual species to antibiotic treatment within a community can be opposite to that in isolation, with sensitive species being able to grow and tolerant species being inhibited in the presence of antibiotic [77]. Given that many chronic infections that require long term, repeat antibiotic treatments — such as infections of the CF lung, urinary tract and diabetic wounds — are caused by polymicrobial communities, it is important to start considering how the composition of bacterial communities modulates not only disease severity but also the success of antibiotic treatment outcomes [15, 78].

Culture-independent methods, such as 16S rRNA sequencing and shotgun metagenomics, are providing an increasingly detailed understanding of the composition of microbiota during infections. These methods have allowed for the tracking of community structure and species richness within individual CF patients through time, across infection types and during antibiotic treatment [79, 80], provided detailed understanding of the response of host microbiota during *Clostridium difficile* infection [81] and directly identified resistance genes during polymicrobial urinary tract [82] and bone and joint infections [83]. As our ability to identify and track resistance determinants within microbial communities improves, the prospect of using clinical metagenomics to design personalised drug regimens becomes more appealing [84]. Despite these advances, we still face a major challenge in identifying which species are interacting within microbial communities and how these may alter both disease progression and the outcomes of antimicrobial treatment. In most cases the specific interactions during polymicrobial infection, or between focal pathogens and the resident commensal communities, remain unknown. To incorporate multi-species interactions into future treatment design requires identification of not only the species present, but also the interactions between them.

How can improved knowledge on microbial ecology be translated into more effective treatment of polymicrobial infections? A detailed and personalised understanding of the microbial taxa present during polymicrobial infections, along with the interactions between them, may facilitate new approaches to antibiotic resistance [74]. Resistance mechanisms that inactivate antibiotics have been the target of inhibitory compounds, such as the co-treatment of β-lactams with β-lactamase inhibitors [85] and such treatments are commonly used in an attempt to overcome the evolution of resistance within focal pathogen strains. In addition, these interventions may be effective against cooperative antibiotic inactivation mechanisms of other members of multi-species communities that are protecting focal pathogens from treatment. However, it is not clear how communities affect the evolution of resistance towards enzyme inhibitors [86]. Other cooperative virulence traits such as EPS production are also vulnerable to perturbation; inhibition of EPS production in *S. enterica* combined with antibiotic treatment proved to be an evolutionarily robust strategy to decrease collective tolerance and reduce the probability of resistance evolution in vitro [87]. The potency of such interventions targeting cooperative traits within polymicrobial biofilms is yet to be investigated. More combinatorial approaches targeting the disruption of interactions that elevate tolerance may be discovered as we gain a greater understanding of the intricacies of interaction networks shaping collective tolerance within bacterial communities.

## Conclusion

Bacteria typically coexist in complex multi-species communities and the interactions within these communities can dictate how bacteria respond to antibiotic exposure. This has important clinical, ecological and environmental consequences, altering levels of tolerance, the selection of resistance and the trajectory of resistance evolution. Conventionally, conclusions about the antibiotic susceptibility of a pathogen are drawn from pure culture measurements of cells in a generally homogenous state. Such information may be adequate for the treatment of infections caused by a single strain. However, it may not be informative of the antibiotic susceptibility of a focal pathogen during polymicrobial infection or embedded within a commensal microbial community. We propose that to effectively design antimicrobial stewardship for pathogens residing in communities we need to view antibiotic resistance as an emergent property that arises as a result of combined effects of antibiotic exposure and microbial interactions within communities.

While understanding AMR in a community context is challenging given the diversity and complexity of microbial interaction networks, it can be achieved through the careful combination of complementary approaches: (1) antibiotic susceptibility testing, where appropriate, should be conducted upon communities in addition to single-cell cultures because resistance is determined by the interactions taking place within that specific community; (2) This should be combined with analyses exploring the consequences of antibiotic treatments on community structure and functioning, which could further change community susceptibility to antibiotics during long term or repeated treatments which are common in chronic, polymicrobial infections; (3) The evolutionary responses to antibiotic treatment should not only focus on the focal pathogen but also upon the wider community in which it resides, as the community is likely to alter the selection dynamics of resistance or could act as reservoir for antibiotic genes. While the effect of ecological context upon antibiotic resistance is important in clinical environments, it should also be addressed in natural microbial communities that are regularly exposed to antibiotic residues through contaminated manure, sewage and wastewater. Moreover, it will also be necessary to move beyond bacterial interactions to also consider the role of multi-kingdom interactions in AMR in the future. Studies increasingly highlight the importance of microbial ecology in determining tolerance, resistance and evolutionary responses to antibiotic treatment, further consideration and quantification of interactions within bacterial community should be paramount.
